# Development of an Innovative Process for High-Temperature Fruit Juice Extraction Using a Novel Thermophilic Endo-Polygalacturonase From *Penicillium oxalicum*

**DOI:** 10.3389/fmicb.2020.01200

**Published:** 2020-06-12

**Authors:** Zhong Cheng, Liang Xian, Dong Chen, Jian Lu, Yutuo Wei, Liqin Du, Qingyan Wang, Yunlai Chen, Bo Lu, Dewu Bi, Zhikai Zhang, Ribo Huang

**Affiliations:** ^1^College of Mechatronic and Quality Technology Engineering, Nanning University, Nanning, China; ^2^State Key Laboratory for Conservation and Utilization of Subtropical Agro-Bioresources, College of Life Science and Technology, Guangxi University, Nanning, China; ^3^National Engineering Research Center for Non-food Biorefinery, State Key Laboratory of Non-food Biomass Enzyme Technology, Guangxi Key Laboratory of Biorefinery, Guangxi Academy of Sciences, Nanning, China; ^4^School of Environment and Life Science, Nanning Normal University, Nanning, China

**Keywords:** fruit juice, high-temperature depectinization, simultaneous heat-treatment and depectinization, thermophilic endo-polygalacturonase, high-level expression, *Penicillium oxalicum*

## Abstract

Efficient and cost-effective production of thermophilic endo-polygalacturonase is desirable for industrial fruit juice production, because its application could shorten the processing time and lower the production cost, by eliminating the separate step of pectin degradation. However, no endo-polygalacturonase that both functions well at sufficiently high temperature and can be manufactured economically, has been reported previously. In this study, the cDNA encoding a thermophilic endo-polygalacturonase from *Penicillium oxalicum* CZ1028, was cloned and over-expressed in *Pichia pastoris* GS115 and *Escherichia coli* BL21(DE3). The recombinant proteins PoxaEnPG28B-Pp (from *P. pastoris*) and PoxaEnPG28B-Ec (from *E. coli*) were isolated and purified. PoxaEnPG28B-Pp was sufficiently thermostable for potential industrial use, but PoxaEnPG28B-Ec was not. The optimal pH and temperature of PoxaEnPG28B-Pp were pH 5.0 and 65°C, respectively. The enzyme had a low *K*_m_ of 1.82 g/L and a high *V*_max_ of 77882.2 U/mg, with polygalacturonic acid (PGA) as substrate. The performance of PoxaEnPG28B-Pp in depectinization of papaya, plantain and banana juices at 65°C for 15 min was superior to that of a reported mesophilic endo-polygalacturonase. PoxaEnPG28B-Pp is the first endo-polygalacturonase reported to show excellent performance at high temperature. An innovative process, including a step of simultaneous heat-treatment and depectinization of fruit pulps with PoxaEnPG28B-Pp, is reported for the first time.

## Introduction

Fruit juices are popular beverages in most parts of the world, with many different flavors, that contain abundant nutrients and bioactive components, and meet the personal taste preferences of many consumers (Ahmad and Chwee, 2008). The industrial production of fruit juices includes four steps. First, in the raw material treatment step, fresh fruits are peeled, cut and pulped. Second, comes the heat-treatment step, whereby fruit pulps are heated at 65°C for 15 min to: (i) inactivate the enzymes that would reduce the quality of the juice (e.g., polyphenol oxidase) (Illera et al., 2018), (ii) accelerate the disruption of the cell structure, to improve the release of cell contents (e.g., polyphenols and anthocyanin pigments) (Marszałek et al., 2017) and (iii) reduce the risk of microbial contamination (Ahmad and Chwee, 2008). In the third, depectinization step, industrial pectinase is added to hydrolyze pectin at 50°C for 1 h. In the fourth and final step, the pulp is pressed and the resulting crude fruit juice is clarified, filtered, pasteurized, and packaged (Lozano, 2006).

Industrial pectinases applied in the fruit juice industry have two drawbacks. One drawback is that they are stable only at relatively low temperatures. Industrial pectinases are mainly sourced from *Aspergillus niger* strains. Their optimal temperatures are 40–50°C, and they are stable only below 45°C (Singh and Rao, 2002; Wang et al., 2017). The low catalytic efficiency and inferior thermal stability of industrial pectinases at 65°C, requires the heat-treatment step (65°C for 15 min) and the depectinization step (at 50°C for 1 h) to be carried out separately (Lozano, 2006). The application of thermophilic pectinases, which were stable and highly active, as part of the heat-treatment step, could eliminate the separate depectinization step, thereby shortening the processing time and lowering equipment costs for fruit juice manufacturing (Kashyap et al., 2001).

Another drawback of industrial *A. niger* pectinases is the presence of contaminating pectinesterase (Kent et al., 2016). Pectinesterases remove the methoxyl groups from the main chain of pectin, generating undesirable, toxic methanol (Alkorta et al., 1998). The best currently available pectinase for the fruit juice industry is endo-polygalacturonase (endo-PGase, EC 3.2.1.15), because it randomly cleaves the α-1,4 glycosidic bonds between two unmethylated residues, leading to rapid degradation of the cell wall, and does not generate methanol (Jayani et al., 2005). Thus, a thermophilic endo-PGase would be the ideal bio-catalyst for a simultaneous heat-treatment and depectinization step.

Many endo-PGases have been described and their performance in fruit juice production was characterized, however, because of their low thermostability, their effects were tested at temperatures much lower than 65°C. For example, 30°C (Tounsi et al., 2016), 40°C (Pan et al., 2015), and 45°C (Cheng et al., 2017) were reported. Moreover, the previously reported endo-PGases were tested on only one type of fruit (Yang et al., 2011; Tu et al., 2013; Tounsi et al., 2016; Wang et al., 2017), however, the inherent characteristics of fruit juices (e.g., pH, pectin content, enzyme inhibitors, etc.) vary substantially. Therefore, testing the performance of an endo-PGase on different fruit juices is of great practical importance for the industry (Cheng et al., 2017).

Papaya (Aldemita et al., 2015), plantain (aka, cooking banana) (Chandler, 1995) and banana (Chandler, 1995) are well-known fruits, possessing high nutritional value and unique flavors and fragrances (Oliveira and Vitória, 2011; Megías-Pérez et al., 2014). However, the highly active metabolism in harvested papaya (Karakurt and Huber, 2003), plantain (Akubor, 2005) and banana (Hakim et al., 2013) can lead to rapid ripening to the point of over-ripeness, which makes the fruits more susceptible to mechanical damage and microbial spoilage (Ali et al., 2004). The high enzymatic activity causes losses of 10–30% of annual global yields in the storage and transport of papaya, plantain and banana crops (Paulla et al., 1997; Hakim et al., 2013). Production of fruit juices [including fruit wines, using fruit juices as raw material (Samoticha et al., 2017; Gaspar et al., 2019)] is an efficient method to generate economic benefits by reducing the loss of perishable fruits and adding value (Lozano, 2006).

We previously reported a thermo-active endo-PGase EPG4, purified from a newly isolated *Penicillium oxalicum* CZ1028, which had optimum enzymatic activity at 60–70°C (Cheng et al., 2016). An endo-PGase applicable to combined high-temperature extraction/depectinization of fruit juices from multiple kinds of fruit would be very attractive to the industry (Kashyap et al., 2001). However, the enzyme activity of endo-PGase in the culture broth of *P. oxalicum* CZ1028 was only 39.9 U/ml. This low yield of EPG4 would severely limit its potential for industrial application.

In this study, the cDNA encoding EPG4 was cloned and overexpressed in *Escherichia coli* and *Pichia pastoris* GS115. The recombinant proteins PoxaEnPG28B-Ec and PoxaEnPG28B-Pp were purified and characterized. PoxaEnPG28B-Pp was applied to laboratory-scale papaya, plantain and banana juice production at 65°C for 15 min and the performance was excellent. To our knowledge, this is the first report of a successful fruit juice process involving a combined heat-treatment and depectinization step.

## Materials and Methods

### Chemicals and Reagents

Chemicals and reagents have been listed in the Supplementary Material (see section “Chemicals and Reagents”).

### Strains and Plasmid

The endo-PGase coding cDNA was cloned from *P. oxalicum* CZ1028 (CGMCC 3.15505) (Cheng et al., 2016). The culture of *P. oxalicum* CZ1028 was as described previously (Cheng et al., 2017). *E. coli* DH5α was used for construction of recombinant plasmids pET22b(+)-*PoxaEnPG28B* and pPIC9K-*PoxaEnPG28B*. Vectors pET22b(+) and pPIC9K were used for gene cloning and expression in *E. coli* BL21(DE3) and *P. pastoris* GS115, respectively. *E. coli* strains and *P. pastoris* GS115 were cultured as reported previously (Cheng et al., 2017).

### Gene Cloning and Heterologous Expression

Two amino acid sequences, EWSGPLLQISGK and WWDGEGSNGGK, obtained from the Matrix-Assisted Laser Desorption Ionization Time-of-Flight mass spectrometry of the previously reported enzyme EPG4 (purified from the culture broth of *P. oxalicum* CZ1028) (Cheng et al., 2016), were used as tags to search the GenBank protein data base, using the BLASTP online server^1^ (Xian and Feng, 2018). Both tags were found to be harbored by a putative protein of *P. oxalicum* 114-2 (GenBank accession no. EPS29213), thus the cDNA encoding EPS29213 was found. Since the genome of different *P. oxalicum* strains are very similar (Xian et al., 2016; Cheng et al., 2017), specific primers were designed according to the gene sequence encoding the putative protein EPS29213, to amplify the cDNA fragment without the signal peptide-encoding region (Supplementary Table S1). The amplified cDNA was inserted into vectors pET22b(+) and pPIK9K. Gene expression by *E. coli* was achieved by using strain BL21(DE3), and the expression was induced by 0.5 mM isopropylthio-galactoside (at 16°C for 12 h) (Xian et al., 2019). Gene expression by *P. pastoris* was carried out as reported previously (Cheng et al., 2017). Detailed methods have been provided in the Supplementary Material (see section “Gene Cloning and Heterologous Expression”).

### Enzyme Assay

The reaction system contained 0.05 ml of appropriately diluted enzyme and 0.45 mL of 0.5% PGA in 0.1 M citric acid–Na_2_HPO_4_ buffer (pH 5.0). After 15 min incubation at 65°C, the reaction was terminated by adding 1 ml of 3,5-dinitrosalicylic acid reagent (Miller, 1959) and was then boiled in water for 10 min. After the mixture had cooled to room temperature, the absorbance of the mixture was measured at 540 nm, and the concentration of the released reducing sugar was calculated using a standard curve constructed with D-galacturonic acid as the standard. One unit of PGase activity was defined as the liberation of 1 μmol of reducing sugar from the enzyme-catalyzed reaction system, in 1 min.

### Protein Purification and Determination

The recombinant protein PoxaEnPG28B-Ec was isolated and purified from the culture medium of *E. coli* BL21(DE3) by using Ni^2+^ affinity chromatography at 4°C (Xian et al., 2019). The recombinant protein PoxaEnPG28B-Pp in culture medium of *P. pastoris* GS115, was concentrated by ultrafiltration, then purified by size-exclusion chromatography on a HiLoad 16/600 superdex 75 column (GE Co., Ltd., Uppsala, Sweden) (Supplementary Material see section “Purification of the Recombinant Protein”). The purity and molecular weight of the enzyme were checked using sodium dodecyl sulfate polyacrylamide gel electrophoresis (SDS-PAGE) with a 5% (w/v) stacking gel and a 10% (w/v) separating gel (Laemmli, 1970). The protein concentration was determined using the Bradford method with bovine serum albumin as standard (Bradford, 1976). The purified enzymes were desalted and used for biochemical characterization and depectinization of fruit pulps.

### Biochemical Properties of the Purified Endo-PGases

Biochemical properties of the purified endo-PGase, effects of pH and temperature on enzyme activity and stability, substrate specificity and kinetic parameters of the enzyme, and the effects of chemicals on enzyme activity were determined using the methods provided in Supplementary Material (see section “Biochemical Properties of the Purified Endo-PGase PoxaEnPG28B”).

### Simultaneous Heat-Treatment and Depectinization of Fruit Pulps at 65°C for 15 Min

In this study, the purified thermo-active endo-PGase PoxaEnPG28B and a previously reported mesophilic endo-PGase PoxaEnPG28A (Cheng et al., 2017) were compared by application to fruit juice production. For evaluation of the performance of these enzymes at high temperature, they were employed in the heat-treatment step (at 65°C for 15 min) instead of the traditional depectinization step (at 50°C for 1 h), and the effects on the resulting fruit juices were analyzed.

Fresh papaya [*Chaenomeles sinensis* (Thouin) Koehne], banana (*Musa nana* Lour) and plantain (*Musa basjoo* Siebold) fruits were purchased from a local market. Fruit pulps were obtained by cutting the peeled fruit into pieces, adding an equal weight of distilled water, then blending using a lab mixer. Enzyme dosages of 0.01, 0.02, or 0.04 mg protein/kg pulp were added to 25 g of each pulp to degrade the pectin at 65°C for 15 min. Then, the pulps were centrifuged at 2,370 × *g* for 10 min at 25°C and the supernatant was collected for analysis. The viscosity of juice was measured using an Ostwald’s viscometer and the viscosity reduction was calculated as described previously (Celestino et al., 2006). The clarity was determined by assaying the light transmittance of the juice at 660 nm. The values for fruit pulp without enzyme addition were defined as 100%, and other values were calculated relative to that.

### Sequence Analysis and Nucleotide Sequence Accession Number

The methods used for analysis of DNA and amino acid sequences have been provided in Supplementary Material (see section “Sequence Analysis”). The nucleotide sequence of the gene encoding PoxaEnPG28B has been deposited in the GenBank database under the accession no. KX236189.

## Results

### Cloning and Heterologous Expression of the Putative Gene and Purification of the Recombinant Proteins

The amino acid sequences (EWSGPLLQISGK and WWDGEGSNGGK) of the thermo active endo-PGase EPG4 were found to be identical to two inner sequences (Glu^97^ to Lys^108^ and Trp^127^ to Lys^137^) of a putative protein from *P. oxalicum* 114-2 (GenBank accession no. EPS29213). The Met^1^ to Ala^19^ region of the putative protein EPS29213 was predicted to form a signal peptide, thus the cDNA fragment encoding Ser^20^ to Ser^380^ of the putative protein EPS29213 was cloned into vectors pPIK9K and pET22b(+) generating plasmid pPIC9K-*PoxaEnPG28B* and pET22b(+)-*PoxaEnPG28B*, respectively.

After transformation of pPIC9K-*PoxaEnPG28B* into the genome of *P. pastoris* GS115, a recombinant strain expressing PGase activity of 1107.6 U/ml in culture broth was obtained. In contrast, the PGase activity in culture broth of *E. coli* BL21(DE3) was only 7.7 U/ml.

**FIGURE 1:**
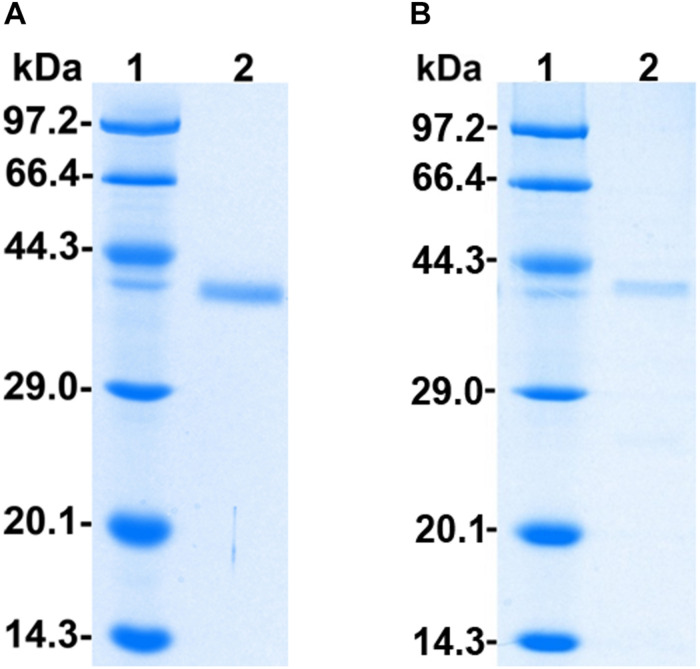
SDS-PAGE analysis of PoxaEnPG28B-Pp and PoxaEnPG28B-Ec. Lane 1 **(A,B)** protein molecular weight ladder (3450Q, from Takara, Co., Ltd., Dalian, China); lane 2, The purified recombinant proteins PoxaEnPG28B-Pp **(A)** and PoxaEnPG28B-Ec **(B)**.

The recombinant protein PoxaEnPG28B-Pp was purified to electrophoretic homogeneity by a simple two-step procedure of ultrafiltration followed by size-exclusion chromatography with a final yield of 16.6% (Supplementary Table S2). The purified PoxaEnPG28B-Pp showed a single band at approximately 38.0 kDa on an SDS-PAGE gel (Figure 1A). The recombinant protein PoxaEnPG28B-Ec was purified in one step, with a final yield of 60.3%, and gave a single band of about 38.0 kDa on an SDS-PAGE gel (Figure 1B).

### Characterization of the Recombinant Enzymes PoxaEnPG28B-Pp and PoxaEnPG28B-Ec

**FIGURE 2:**
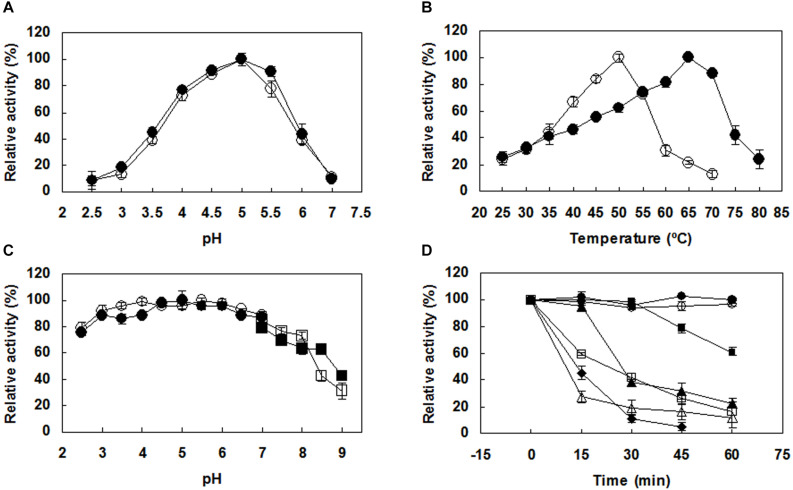
Effects of pH and temperature on the enzyme activity and stability of the purified PoxaEnPG28B-Pp and PoxaEnPG28B-Ec. **(A)** Effect of pH on enzyme activities of PoxaEnPG28B-Pp (∙) and PoxaEnPG28B-Ec (∘). **(B)** Effect of temperature on enzyme activities of PoxaEnPG28B-Pp (∙) and PoxaEnPG28B-Ec (∘). **(C)** Effect of pH on enzyme stability of PoxaEnPG28B-Pp (∙ and ■) and PoxaEnPG28B-Ec (∘ and □). **(D)** Effect of temperature on enzyme stability. For PoxaEnPG28B-Pp, temperatures were 50°C (∙), 55°C (■), 60°C (▲), and 65°C (◆), respectively. For PoxaEnPG28B-Ec, temperatures were 35°C (∘), 40°C (□), and 45°C (△), respectively. Error bars present the standard deviation of three repeats.

Both PoxaEnPG28B-Pp and PoxaEnPG28B-Ec showed optimal activity at pH 5.0 (Figure 2A). PoxaEnPG28B-Pp showed maximal enzyme activity at 65°C and retained 88% of maximal activity at 70°C (Figure 2B). The optimum temperature of PoxaEnPG28B-Ec was 50°C (Figure 2B). Both PoxaEnPG28B-Pp and PoxaEnPG28B-Ec retained more than 85% residual enzyme activity over the pH range 3.0–7.0 (Figure 2C). After incubation for 1 h at 50°C or 55°C, PoxaEnPG28B-Pp retained 100 and 60% enzyme activity, respectively (Figure 2D). PoxaEnPG28B-Pp retained 45% of its enzyme activity when it was incubated at 65°C for 15 min (Figure 2D). PoxaEnPG28B-Ec retained 100% enzyme activity only after incubation at no more than 35°C for 1 h (Figure 2D). At an incubation temperature of 40°C for 1 h, PoxaEnPG28B-Ec lost 80% of its enzyme activity (Figure 2D).

When assayed under optimal conditions (pH 5.0 and 65°C), PoxaEnPG28B-Pp showed specific activities of 31974.3 U/mg on PGA (100%), 10807.3 U/mg on citrus pectin (33.8%) and 6075.1 U/mg on apple pectin (19.0%). The *K*_m_ and *V*_max_ values of PoxaEnPG28B-Pp were calculated as 1.82 g/L and 77882.2 U/mg, respectively (Supplementary Figure S1).

The metal ions, K^+^, Mg^2+^ and Na^+^, which are present in many fruits, did not affect the enzyme activity of PoxaEnPG28B-Pp at concentrations of 1 mM, or 2 mM (Table 1). Heavy metal ions, Ba^2+^, Co^2+^, Cu^2+^, Mn^2+^, and Zn^2+^, which have been usually reported to inhibit the enzyme activities of other endo-PGases, also partially inhibited the enzyme activity of PoxaEnPG28B-Pp. Ca^2+^ at a concentration of 2 mM slightly inhibited the enzyme activity of PoxaEnPG28B-Pp, but 97.1% enzyme activity was retained in the presence of 1 mM Ca^2+^. At 2 mM Fe^2+^, the enzyme activity of PoxaEnPG28B-Pp was partially inhibited.

**TABLE 1:** Effects of metal ions on the enzyme activity of PoxaEnPG28B-Pp.

Metal salts	Relative activity^a^ (%)	
	Concentration (mM)	
	1	2
Control	100.0 ± 0.8	100.0 ± 0.3
BaCl_2_	101.6 ± 2.9	92.5 ± 0.9
CaCl_2_	97.1 ± 3.0	90.9 ± 0.9
CoCl_2_	86.4 ± 1.3	67.7 ± 0.4
CuCl_2_	14.1 ± 3.1	9.5 ± 5.3
FeCl_2_	99.6 ± 1.9	76.5 ± 1.0
KCl	105.2 ± 2.1	99.4 ± 0.8
MgCl_2_	101.4 ± 1.1	98.1 ± 1.5
MnCl_2_	49.7 ± 2.0	39.8 ± 2.1
NaCl	103.9 ± 1.0	99.8 ± 1.1
ZnCl_2_	93.8 ± 0.1	76.0 ± 2.6

*^*a*^All experiments were performed in triplicate and mean values are presented. The experiments were repeated three times and similar results were obtained.*

### Application of PoxaEnPG28B-Pp in High-Temperature Fruit Juice Extraction

At an enzyme dosage of 0.04 mg protein/kg pulp, PoxaEnPG28B-Pp reduced the viscosity of papaya juice by 42.5%, increased the light transmittance by 43.0% and increased the yield by 23.7% (Table 2). PoxaEnPG28B-Pp reduced the viscosity of banana juice by 67.4%, increased the light transmittance by 36.1% and increased the yield by 4.9% (Table 2), and reduced the viscosity of plantain juice by 81.3%, increased the light transmittance by 47.3% and increased the yield by 108.4% (Table 2).

**TABLE 2:** Application of PoxaEnPG28B-Pp in fruit juices production at 65°C.

	Enzyme		Increment	
	dosage	Reduction in	of light	Increment of
Fruits	(mg/kg pulp)	viscosity^a^ (%)	transmittace^a^ (%)	yield^a^ (%)
Papaya	0.01	20.1 ± 1.4	40.4 ± 3.6	20.4 ± 0.8
	0.02	29.6 ± 1.9	41.2 ± 3.3	21.6 ± 1.3
	0.04	42.5 ± 2.9	43.0 ± 2.9	23.7 ± 1.5
Banana	0.01	56.1 ± 1.9	9.4 ± 0.6	3.8 ± 0.4
	0.02	64.2 ± 1.1	21.0 ± 1.4	4.1 ± 0.2
	0.04	67.4 ± 3.7	36.1 ± 2.9	4.9 ± 0.5
Plantain	0.01	71.2 ± 3.6	40.8 ± 3.0	102.8 ± 4.8
	0.02	76.6 ± 4.7	46.8 ± 1.5	105.6 ± 5.3
	0.04	81.3 ± 4.1	47.3 ± 2.3	108.4 ± 4.1

*^*a*^All experiments were performed in triplicate and mean values were presented. The experiments were repeated three times and similar results were obtained.*

In the comparative experiment, using an enzyme dosage of 0.04 mg protein/kg pulp, PoxaEnPG28A reduced the viscosity of papaya juice by 6.6%, increased the light transmittance by 23.2% and increased the yield by 10.0%; reduced the viscosity of banana juice by 35.6%, increased the light transmittance by 5.1% and increased the yield by 2.7%; and reduced the viscosity of plantain juice by 68.7%, increased the light transmittance by 34.0% and increased the yield by 53.5% (Supplementary Table S3).

## Discussion

### Cloning and Heterologous Expression of the Putative Gene and Purification of the Recombinant Proteins

The amino acid sequence encoded by the amplified product was identical to the putative protein EPS29213, and it shared the highest identity (81%) with a reported endo-PGase PG1 from *Penicillium occitanis* (Genbank accession no. ABS50231) (Tounsi et al., 2016). The phylogenetic tree also showed a very close evolutionary relationship between them (Supplementary Figure S2). Cys^58^ to Ser^380^ of the encoded protein was predicted to form a catalytic domain belonging to the glycoside hydrolase family 28. By alignment with a site-directed mutated and functionally analyzed endo-PGase (Cho et al., 2001), conserved amino acids Asp^195^, Asp^216^, Asp^217^, and His^238^ of the encoded protein were predicted to play a crucial role in the catalytic function, whereas Arg^274^ and Lys^276^ were predicted to be involved in substrate binding (Supplementary Figure S3). Four strictly conserved disulphide bridges were predicted to be formed between Cys^40^ and Cys^58^, Cys^218^ and Cys^234^, Cys^346^ and Cys^351^, and Cys^370^ and Cys^379^, respectively.

Since the enzyme activity of the culture medium of *E. coli* was low and the enzyme was not thermostable, *E. coli* was not suitable for industrial production of this enzyme. The yield of the recombinant enzyme from *P. pastoris* GS115 (1107.6 U/ml) was 28 times that of the natural enzyme EPG4 from *P. oxalicum* CZ1028 (39.9 U/ml) (Cheng et al., 2016), indicating that the recombinant over-expression by *P. pastoris* GS115 was successful. The PGase activity in the culture medium of the recombinant *P. pastoris* GS115 was higher than those of endo-PG I produced by *P. pastoris* (6.2 U/ml) (Yuan et al., 2011), endo-PGA1 produced by *P. pastoris* (50 U/ml) (Yang et al., 2011), PG1 produced by *Saccharomyces cerevisiae* (50 U/ml) (Rojas et al., 2011) and PG7fn produced by *P. pastoris* (678.1 U/ml) (Tu et al., 2014). Purified natural endo-PGases showing excellent enzymatic characteristics were reported (Miyairi et al., 1994; Tounsi et al., 2016); however, high-yield recombinant production of neither of these enzymes has been reported. The low yields of these enzymes from the source strains would seriously limit their industrial application. The high-yield recombinant production of PoxaEnPG28B-Pp (1107.6 U/ml) should facilitate the industrial application of this enzyme.

The molecular weights of the purified PoxaEnPG28B-Pp and PoxaEnPG28B-Ec were in agreement with the predicted molecular weight of 37.14 and 37.10 kDa, respectively, and were similar to some endo-PGases reported in the literature (i.e., 35–40 kDa) (Yuan et al., 2011; Tu et al., 2013; Cheng et al., 2017).

### Characterization of the Recombinant Enzymes PoxaEnPG28B-Pp and PoxaEnPG28B-Ec

Most previously reported endo-PGases have an optimal pH too far away from the mildly acidic pH of fruit juice to be useful industrially (Pařenicová et al., 1998; Belarbi et al., 2000; Sieiro et al., 2009; Rojas et al., 2011; Yang et al., 2011; Yuan et al., 2011; Pan et al., 2015; Tu et al., 2018). PoxaEnPG28B-Pp and PoxaEnPG28B-Ec (pH optimum 5.0) should be more suitable for production of tropical and subtropical fruit juices (papaya, plantain, and banana, etc.), since they are mildly acidic (pH 4.5–6.0) (Grassin and Coutel, 2009). Both PoxaEnPG28B-Pp and PoxaEnPG28B-Ec retained more than 85% enzyme activity after incubation at a pH range of 3.0–7.0. The stable pH range of these two enzymes covered the pH range of most fruits (4.0–6.5), and was broader than that of most reported endo-PGases, such as NfPG I (pH 6.0), NfPG II (pH 3.0) and NfPG III (pH 3.5) (Pan et al., 2015), PG1 (pH 4.0–7.0) (Tounsi et al., 2016), and endo-PG I (pH 4.0–6.0) (Yuan et al., 2011).

The optimal temperature of PoxaEnPG28B-Pp was similar to the natural enzyme EPG4 (Cheng et al., 2016), and it was markedly higher than that previously reported for most endo-PGases (Lang and Looman, 1995; Belarbi et al., 2000; Xiao et al., 2008; Yang et al., 2011; Yuan et al., 2011; Tu et al., 2013; Pan et al., 2015; Cheng et al., 2017; Carrasco et al., 2019; Tanaka et al., 2019). The thermostability of PoxaEnPG28B-Pp was superior to PoxaEnPG28A (Cheng et al., 2017), NfPG I, NfPG II (Pan et al., 2015), endo-PG I (Tu et al., 2013), and four PGases from *S. cerevisiae* strain SCPP (Belarbi et al., 2000).

The thermostability of PoxaEnPG28B-Ec was inferior to both PoxaEnPG28B-Pp and the natural enzyme EPG4 (Cheng et al., 2016). *P. pastoris* and *E. coli* are widely used as hosts for heterologous gene expression, however, not all the recombinant proteins can be correctly folded, since the protein folding system may differ between species (Gasser et al., 2008). Improper folding of recombinant protein may reduce the yield (Gasser et al., 2008), lower the catalytic efficiency (Blum et al., 2000) and affect enzymatic characteristics of the recombinant enzyme (Sriyapai et al., 2011). Since PoxaEnPG28B-Ec was heat-labile, it was not suitable for high temperature application and was not further studied.

The higher specific activity of PoxaEnPG28B-Pp on PGA, than on citrus, or apple pectin indicated that esterification of PGA hindered enzymatic hydrolysis by PoxaEnPG28B-Pp (Tu et al., 2013; Tounsi et al., 2016; Cheng et al., 2017).

Previous studies reported *K*_m_ values of endo-PGases ranging from 0.32 to 19.5 g/L (Singh and Rao, 2002; Yang et al., 2011; Yuan et al., 2011; Tu et al., 2013; Pan et al., 2015; Cheng et al., 2017; Wang et al., 2017). The relatively low *K*_m_ value of PoxaEnPG28B-Pp (1.82 g/L) indicated that the enzyme has stronger affinity toward the substrate than most reported endo-PGases. Most endo-PGases have *V*_max_ values below 10,000 U/mg (Singh and Rao, 2002; Yang et al., 2011; Yuan et al., 2011; Wang et al., 2017), whereas PoxaEnPG28B-Pp had markedly higher catalytic efficiency, with a *V*_max_ of 77882.2 U/mg.

### Application of PoxaEnPG28B-Pp in High-Temperature Fruit Juice Extraction

The performance of PoxaEnPG28B-Pp in papaya juice extraction was similar to that of the natural enzyme EPG4 (Cheng et al., 2016), however, the temperature used was higher and the reaction time was shorter than that used previously (Cheng et al., 2016). A mesophilic endo-PG I from *Achaetomium* sp. Xz8 reduced the viscosity of papaya juice by 17.6% and increased the light transmittance by 59.1%, but at a lower temperature of 45°C and for a longer reaction time of 1 h (Tu et al., 2013) (Table 3).

**TABLE 3:** Comparison of enzyme characteristics and performance in fruit juice production of reported endo-PGases.

Enzyme name	Source strain	Optimal temperature	Optimal pH	Tested conditions	Tested fruit juice and performance of endo-PGase	References
PoxaEnPG28B-Pp	*P. oxalicum* CZ1028	65°C^a^	5.0	At 65°C for 15 min	**Papaya:** VR by 42.5 ± 2.9%, LTI by 43.0 ± 2.9%, and YI by 23.7 ± 1.5%; **Banana:** VR by 67.4 ± 3.7%, LTI by 36.1 ± 2.9%, and YI by 4.9 ± 0.5%; **Plantain:** VY by 81.3 ± 4.1%, LTI by 47.3 ± 2.3%, and YI by 108.4 ± 4.1%.	This study
PG1	*Penicillium occitanis* CT1	70°C^b,A^	5.0^B^	At 30°C for 4 h	**Citrus:** YI by 25%.	Tounsi et al., 2016
NfPG II	*Neosartorya fischeri* P1	60°C^c^	4.0	At 40°C for 1 h	**Apple:** VR by 14.3%, LTI by 99%, and YI by 12%; **Strawberry:** VR by 79.4%, LTI by 53.59%, and YI by 9%.	Pan et al., 2015
PoxaEnPG28A	*P. oxalicum* CZ1028	55°C^a^	5.5	At 45°C for 1 h	**Papaya:** VR by 78.36%, LTI by 43.3%, and YI by 32.92%; **Banana:** VR by 31.76%, LTI by 6.2%, and YI by 8.76%; **Plantain:** VR by 23.30%, LTI by −10.3%, and YI by 25.21%.	Cheng et al., 2017
NfPG I	*Neosartorya fischeri* P1	55°C^c^	5.0	At 40°C for 1 h	**Apple:** VR by 10%, LTI by 104%, and YI by 24%; **Strawberry:** VR by 67.6%, LTI by 71.28%, and YI by 6%.	Pan et al., 2015
endo-PGA1	*Bispora* sp. MEY-1	50°C^c^	3.5	At 50°C for 1 h	**Apple:** VR by 7.7% and LTI by 84%.	Yang et al., 2011
endo-PG I	*Achaetomium* sp. Xz8	45°C^c^	6.0	At 45°C for 1 h	**Papaya:** VR by 17.6% and LTI by 59.1%.	Tu et al., 2013
PGA-ZJ5A	*Aspergillus niger* ZJ5	40°C^c^	4.5	At 40°C for 6 h	**Pear:** LTI by nearly 200% and YI by 41.8%.	Wang et al., 2017

*VR, viscosity reduction; LTI, light transmittance increment; YI, yield increment. NM, not mentioned in the reference. ^*a–c*^The reaction times were 15 min^*a*^, 30 min^*b*^, and 10 min^*c*^, respectively. ^*A*^The enzyme activity at 65 and 75°C were not determined, thus the optimum temperature of 70°C is questionable. ^*B*^The enzyme activity at pH 4.5 and pH 5.5 were not determined, thus the optimum pH of 5.0 is questionable.*

PoxaEnPG28B-Pp also functioned well in banana juice extraction (increased the yield by 4.9%). A pectinase preparation from *Sporotrichum thermophile* Apinis increased the yield by only 2% (Kaur et al., 2004), at a lower temperature (55°C) and for a longer reaction time (2 h), than those used in this study. A cold-adapted industrial pectinase preparation (used at 15°C, for 24 h) reduced the viscosity of banana juice by 13–23% (Byaruagaba-Bazirake et al., 2012) (Table 3), much less than achieved with PoxaEnPG28B-Pp (67.4%).

The performance of PoxaEnPG28B-Pp in plantain juice extraction was also excellent. A previously reported mesophilic endo-PGase showed inferior results, reducing the viscosity by 23.30%, increasing the light transmittance by −10.3% and increasing the yield by 25.21% (Cheng et al., 2017) (Table 3). Moreover, the reported mesophilic endo-PGase was tested at a lower temperature (45°C) and for a longer reaction time (1 h) (Cheng et al., 2017) than conditions used for PoxaEnPG28B-Pp.

When PoxaEnPG28A was used under the same conditions as PoxaEnPG28B-Pp, its performance was inferior, both to its previous result, obtained at 45°C for 1 h (Cheng et al., 2017), and to that of PoxaEnPG28B-Pp. These results were attributed to the higher temperature inactivating the enzyme activity of PoxaEnPG28A.

The optimum temperature of 65°C for PoxaEnPG28B-Pp was the highest temperature and 15 min was the shortest reaction time reported for an endo-PGase (Table 3). The industrial fruit juice production process can be shortened and equipment costs can be reduced, by combining the heat-treatment step (at 65°C for 15 min) with the depectinization step (at 50°C for 1 h), but this improvement has never been previously reported (Table 3). The results obtained from the comparative testing indicate that the thermo-active PoxaEnPG28B-Pp was much more suitable than the mesophilic enzyme PoxaEnPG28A, for application to high-temperature fruit pulp depectinization. This is the first practical demonstration of the advantages of thermophilic pectinase in high-temperature fruit juice extraction. Some endo-PGases active at close to 60°C were reported, but their performance in fruit juice extraction at 65°C was not determined (Miyairi et al., 1994; Tounsi et al., 2016). To the best of our knowledge, PoxaEnPG28B-Pp is the first endo-PGase successfully applied in high-temperature fruit juice extraction.

The cell-wall structure, juice pH and cell contents of fruit are dependent on the plant species (Byaruagaba-Bazirake et al., 2012), stage of maturity (Ali et al., 2004) and duration of postharvest storage (Karakurt and Huber, 2003). In previous reports, most endo-PGases were tested for only one type of fruit; however, it is hard to demonstrate that an endo-PGase that functioned well toward one type of fruit, at one stage of maturity and postharvest storage duration, will also function well in other fruits. PoxaEnPG28B-Pp functioned well in juice extraction of three types of fruit, thus suggesting its wide potential application in industrial fruit juice production.

Pectinases produced by *A. niger* represent potential health hazards because they contain pectinesterase, which releases toxic methanol from pectin (Kent et al., 2016). Pectinesterase is expected to be inactivated during the heat-treatment step of fruit-pulp processing (Marszałek et al., 2017; Illera et al., 2018). Application of a pectinesterase-free endo-PGase, however, may still be helpful for production of healthy fruit juice with lower methanol content (Cano et al., 1997). Furthermore, the structure of the fruit cell wall consists of cellulose, hemicellulose and other polysaccharides, and complete degradation of the cell wall requires synergistic action by many bio-macromolecule degrading enzymes (Scheller and Ulvskov, 2010). In a previous report, the yields of banana, grape and apple juices using enzyme blends of pectinase, cellulase, and xylanase were 4-, 3. 33-, and 2.57-fold greater than those obtained using pectinase only, which demonstrates that other cell-wall degrading enzymes can play important roles in fruit juice production (Kaur et al., 2004). The performance of PoxaEnPG28B-Pp may, therefore, be enhanced by combining it with other cell-wall degrading enzymes (Abbès et al., 2011; Guerrero et al., 2016; Comtet-Marre et al., 2017; Toushik et al., 2017; Alexandre et al., 2019; Borchani et al., 2019). Thus, PoxaEnPG28B-Pp could be an excellent substitute for conventional industrial pectinases and have potential applications in enabling a reduced-cost and faster processing treatment, resulting in healthier fruit juices.

## Conclusion

The cDNA encoding a thermo active endo-polygalacturonase was cloned and expressed in both *P. pastoris* GS115 and *E. coli* BL21(DE3). The gene was successfully overexpressed in *P. pastoris* GS115, resulting in a high yield of endo-PGase activity (1107.6 U/ml) in the culture medium; the recombinant protein was highly active and thermostable. The purified recombinant protein PoxaEnPG28B-Pp (from *P. pastoris*) had optimal pH and temperature at pH 5.0 and 65°C, respectively, and it was stable over a pH range of 3.0–7.0. When PoxaEnPG28B-Pp treatment was combined with the heat-treatment step of fruit pulps at 65°C for 15 min, it performed much better than a mesophilic endo-polygalacturonase PoxaEnPG28A. Thus, an innovative process involving a simultaneous heat-treatment and depectinization step was successfully developed, which has great potential for more efficient production of healthier fruit juice.

## Data Availability Statement

The datasets generated for this study can be found in the nucleotide sequence of gene encoding PoxaEnPG28B has been deposited in the GenBank database under the accession no. KX236189.

## Author Contributions

RH, DC, JL, and LX conceived and designed the experiments. ZC performed the major part of experiments and molecular analysis. LX, ZZ, DB, YC, and BL took part in the experiments. ZC, JL, and LX performed bioinformatics analysis. RH, DC, JL, YW, QW, and LD performed data analysis. ZC and LX drafted the manuscript. RH and LX critically reviewed the analysis of experimental data and the contents of the manuscript. All authors read and approved the final manuscript.

## Conflict of Interest

The authors declare that the research was conducted in the absence of any commercial or financial relationships that could be construed as a potential conflict of interest.

## Footnotes

1 http://blast.ncbi.nlm.nih.gov/Blast.cgi
