# Supplementary material for: Development of an Innovative Process for High-Temperature Fruit Juice Extraction Using a Novel Thermophilic Endo-Polygalacturonase From Penicillium oxalicum
Source: Front Microbiol. 2020 Jun 12;11:1200. doi: 10.3389/fmicb.2020.01200 (PMC7303257; doi:10.3389/fmicb.2020.01200)
Supplement: Supplementary file 1 [file Data_Sheet_1.pdf]

Journal: **Frontiers in Microbiology**

Supplementary material for

Development of an innovative process for high-temperature fruit juice extraction using a novel thermophilic endo-polygalacturonase from *Penicillium oxalicum*

Zhong Cheng[1,2,†], Liang Xian[3,†], Dong Chen[3,†], Jian Lu[2,†], Yutuo Wei[2], Liqin Du[2], Qingyan Wang[3], Yunlai Chen[4], Bo Lu[3], Dewu Bi[2,3], Zhikai Zhang[2,3] and Ribo Huang[2,3,*]

[1] *College of Mechatronic and Quality Technology Engineering, Nanning University, Nanning, China*

[2] *College of Life Science and Technology, State Key Laboratory for Conservation and Utilization of Subtropical Agro-bioresources, Guangxi University, Nanning, China*

[3] *National Engineering Research Center for Non-food Biorefinery, State Key Laboratory of Non-food Biomass Enzyme Technology, Guangxi Key Laboratory of Biorefinery, Guangxi Academy of Sciences, Nanning, China*

[4] *School of Environment and Life Science, Nanning Normal University, Nanning, China*

[†]Co-first author: Zhong Cheng, Liang Xian, Dong Chen and Jian Lu contributed equally to this study.

*Corresponding author (Ribo Huang).

E-mail: rbhuang@gxas.cn. Tel.: +86-771-2503982; fax: +86-771-2503908.

Materials and Methods

Chemicals and Reagents

RNA purification reagent (RNAiso Plus), *LA* Taq DNA polymerase, restriction endonucleases, T4 DNA ligase, and protein marker were obtained from TaKaRa Bio Co. Ltd. (Dalian, China). mRNA reverse transcriptase was purchased from TransGen Biotech Co. Ltd. (Beijing, China). Polygalacturonic acid (PGA, de-esterified), citrus pectin (＜26% esterification), apple pectin (50–75% esterification), trigalacturonic acid and D-galacturonic acid were obtained from Sigma-Aldrich Co. Ltd. (Saint Louis, MO, USA). Unless otherwise stated, all other chemicals were of analytical grade and commercially available.

Gene Cloning and Heterologous Expression

Total RNA extraction and mRNA reverse transcription were carried out using a previously reported method (Cheng et al., 2017). Since the genomic sequences of *P. oxalicum* strains are highly similar (Xian et al., 2016), specific primers *PoxaEnPG28BF* (5-′GACC<u>TACGTA</u>TCACCCGTCGCTGAGCCG-3′) (*SnaB* I site was underlined) and *PoxaEnPG28BR* (5-′CGA<u>CCTAGG</u>TCAAGAGCACTTGGTCACACTGG-3′) (*Avr* II site was underlined) were designed to amplify the cDNA fragment without the signal peptide-encoding region. The amplified product was digested by *SnaB* I and *Avr* II and inserted into the vector pPIC9k, and the generated plasmid was named as pPIC9K-*PoxaEnPG28B*. Plasmid pPIC9K-*PoxaEnPG28B* was linearized by *Sac* I and transformed into the genome of *P. pastoris* GS115 for secreted expression using the LiCl method according to the manual instructions supplied by Invitrogen Co. Ltd. The recombinant strains were screened using the method described previously (Cheng et al., 2017), and the strain expressing the highest PGase activity in

the culture broth was chosen for further research.

Purification of the Recombinant Protein

After three days of inducement, the culture broth of the recombinant *P. pastoris* GS115 was centrifuged (11,325×*g*, for 10 min, at 4 °C), and the supernatant was filtrated through a membrane (size of 0.22 μm) followed by concentration using an ultrafiltration membrane with a molecular weight cut-off of 10 kDa (Millipore, Ireland). The concentrated supernatant was applied to size-exclusion chromatography using a HiLoad 16/600 superdex 75 column (GE Co., Ltd., Uppsala, Sweden) and was eluted by 20 mM KH_2PO_4-K_2HPO_4 buffer (pH 6.0) with a flow rate of 1.0 mL/min. The fractions exhibiting PGase activity were applied to sodium dodecyl sulphate polyacrylamide gel electrophoresis (SDS-PAGE), with a 5% (w/v) stacking gel and a 10% (w/v) separating gel (Laemmli, 1970). The protein concentration was determined using the Bradford method, with bovine serum albumin protein as standard (Bradford, 1976).

Biochemical Properties of the Purified Endo-PGase PoxaEnPG28B

The optimal pH of the purified endo-PGase was assayed by determining the enzyme activity at a pH range of 3.0–7.0 and at 50 °C. The optimal temperature of the purified endo-PGase was assayed by determining the enzyme activity at 25–80 °C and at an optimal pH of 5.0. The highest enzyme activity was defined as 100%, and other enzyme activities were calculated as relative values.

The pH stability of the purified endo-PGase was assayed by incubating the enzyme in buffers with different pH values (0.1 M citric acid-Na_2HPO_4 for pH 3.0–7.0 and 0.1 M Tris-HCl for pH 7.0–9.0) at 25 °C for 24 h (without substrate), and then the residual activities were measured. The thermo stability of the purified endo-PGase was assayed by incubating the enzyme (at an optimal

pH of 5.0) at different temperatures for various periods (with substrate), and the residual activities were measured. The enzyme activity of untreated enzyme was defined as 100%, and the residual enzyme activities were calculated as relative values.

In order to determine the substrate specificity, the enzyme activities on 0.5% (W/V) PGA, citrus pectin and apple pectin were tested under optimal pH (pH 5.0) and temperature (65 °C) conditions. The enzyme activity on PGA was defined as 100%, and other enzyme activities were calculated as relative values.

The kinetic parameters of the purified endo-PGase were determined by assaying the specific activities of the enzyme on 0.2-2.0 g/L of PGA at optimal pH (pH 5.0) and temperature (65 °C). The K_m and V_{max} values were obtained by calculation using the Line-weaver Burk plot method.

The effects of chemicals on the enzyme activity of the purified endo-PGase were investigated by adding the metal ions (1 or 2 mM of Na^+, K^+, Mg^{2+}, Ca^{2+}, Mn^{2+}, Ba^{2+}, Co^{2+}, Zn^{2+}, Fe^{2+} or Cu^{2+}) into the reaction system, and measuring the enzyme activities. The enzyme activity without additive was defined as 100%, and other enzyme activities were calculated as relative values.

Sequence Analysis

The signal peptide was predicted using SignalP 4.1 online Server (http://www.cbs.dtu.dk/services/SignalP/). The catalytic domain was predicted using SMART online server (http://smart.embl-heidelberg.de/). The hypothetical molecular weight of proteins was calculated using ExPASy online server (http://web.expasy.org/protparam/). Multiple sequence alignment was carried out using the DNAMAN 6.0 software, and the reported fungal endo-PGases used were PGA (from *Aspergillus aculeatus*, GenBank accession no. 1IA5_A) (Cho et al., 2001), pga II (from *Aspergillus niger*, Genbank accession no. CAA41694) (van Santen et al.,

1999), PoxaEnPG28A (from *Penicillium oxalicum* CZ1028, Genbank accession no. KU366356) (Cheng et al., 2017), pgaE (from *Aspergillus niger* N400, Genbank accession no. O42809) (Pařenicová et al., 1998), PG7fn (from *Thielavia arenaria* XZ7, Genbank accession no. AIZ95162) (Tu et al., 2014), endo-PG I (from *Achaetomium* sp. Xz-8, Genbank accession no. AGR51994) (Tu et al., 2013), CluPG1 (from *Colletotrichum lupine* SHK788, Genbank accession no. 2IQ7) (Bonivento et al., 2008) and ENPG-1 (from *Cryphonectria parasitica*, Genbank accession no. AAB36616) (Gao et al., 1996). Phylogenetic tree was constructed using the Mega software (Tamura et al., 2004). Other reported endo-PGases like endo-PG I (from *Penicillium* sp. CGMCC 1669, Genbank accession no. AEL22832) (Yuan et al., 2011), endo-PGA1 (from *Bispora* sp. MEY-1, Genbank accession no. ADZ99366) (Yang et al., 2011), RePgaA (from *Aspergillus niger* JL-15, Genbank accession no. AGV40780) (Liu et al., 2014), endo-PgaA (from *Aspergillus niger* SC323, Genbank accession no. AJD09825) (Zhou et al., 2015), Epg1-2p (from *Kluyveromyces marxianus* CECT1043, Genbank accession no. AAR84199) (Sieiro et al., 2009), PGA-ZJ5A (from *Aspergillus niger* ZJ5, Genbank accession no. AQT01640) (Wang et al., 2017), PehA (from *Burkholderia cepacia* ATCC 25416, Genbank accession no. AAB46984) (Gonzalez et al., 1997; Massa et al., 2007), endo-PGase (from *Pectobacterium carotovorum*, Genbank accession no. WP_039543807) (Rafique et al., 2016), ThPG1 (from *Trichoderma harzianum* T34, Genbank accession no. CAM07141) (Morán-Diez et al., 2009) were also included for phylogenetic tree construction.

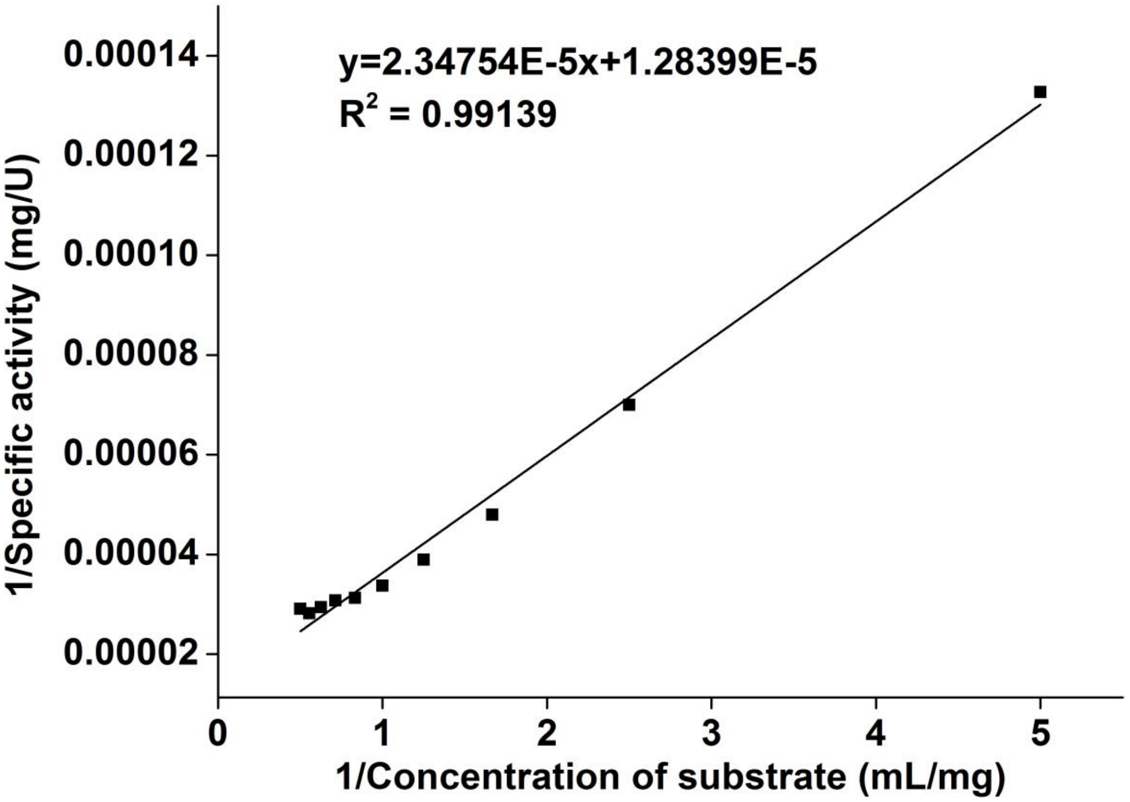

FIGURE S1. The kinetic parameters of the purified PoxaEnPG28B-Pp.

The kinetic parameters of the purified PoxaEnPG28B-Pp were determined by assaying the reaction rates for polygalacturonic acid at concentrations ranging from 0.2–2.0 g/L under the standard assay condition (at pH 5.0, 65 °C and for 15 min) (A)，error bars present the standard deviation of three repeats. The Michaelis constant (*K*m) and the maximum reaction velocity (*V*max) were obtained from the Line-weaver Burk plot (B).

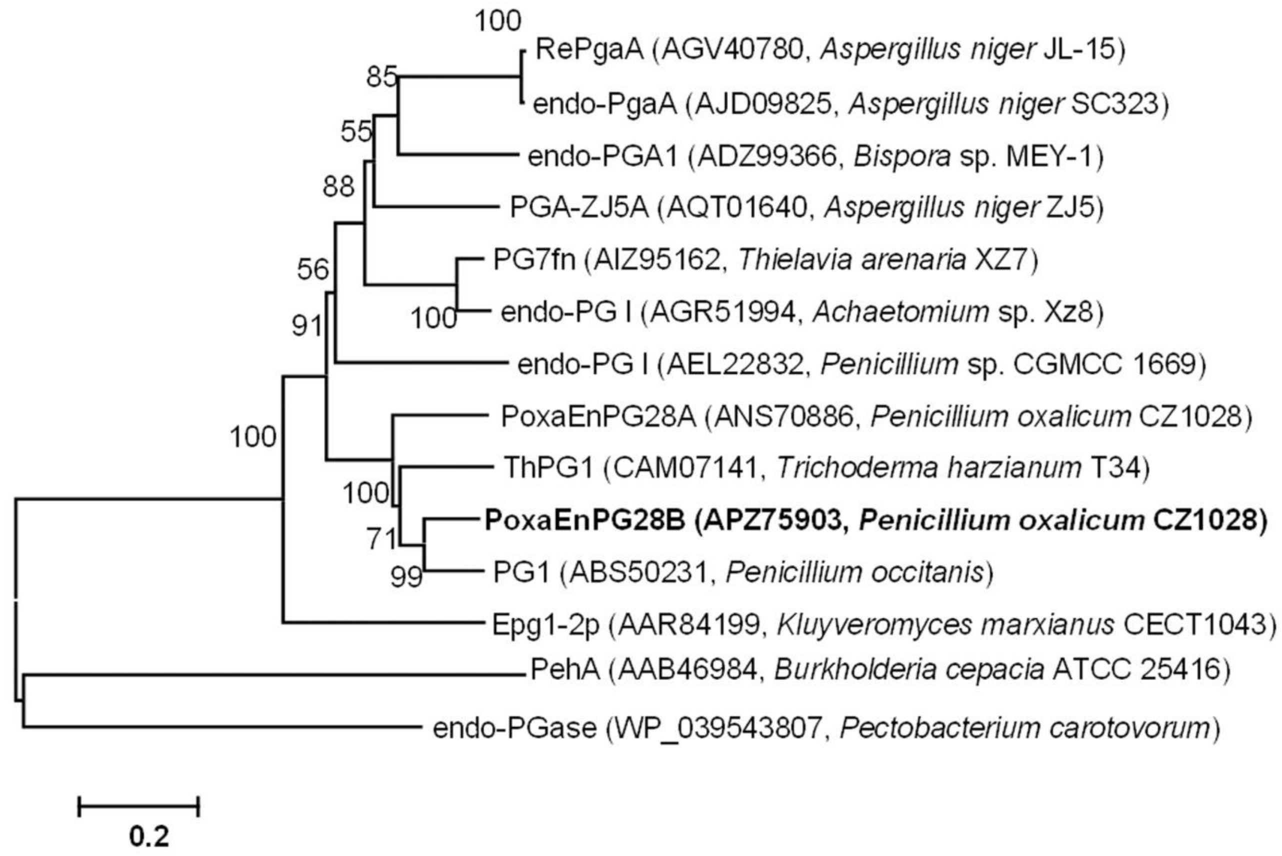

FIGURE S2. Phylogenetic tree of amino acids of PoxaEnPG28B with reported endo-PGases.

Phylogenetic tree of amino acids of PoxaEnPG28B with reported endo-PGases (from fungus, yeast and bacteria) was constructed by using MEGA software. PoxaEnPG28B was boldfaced.

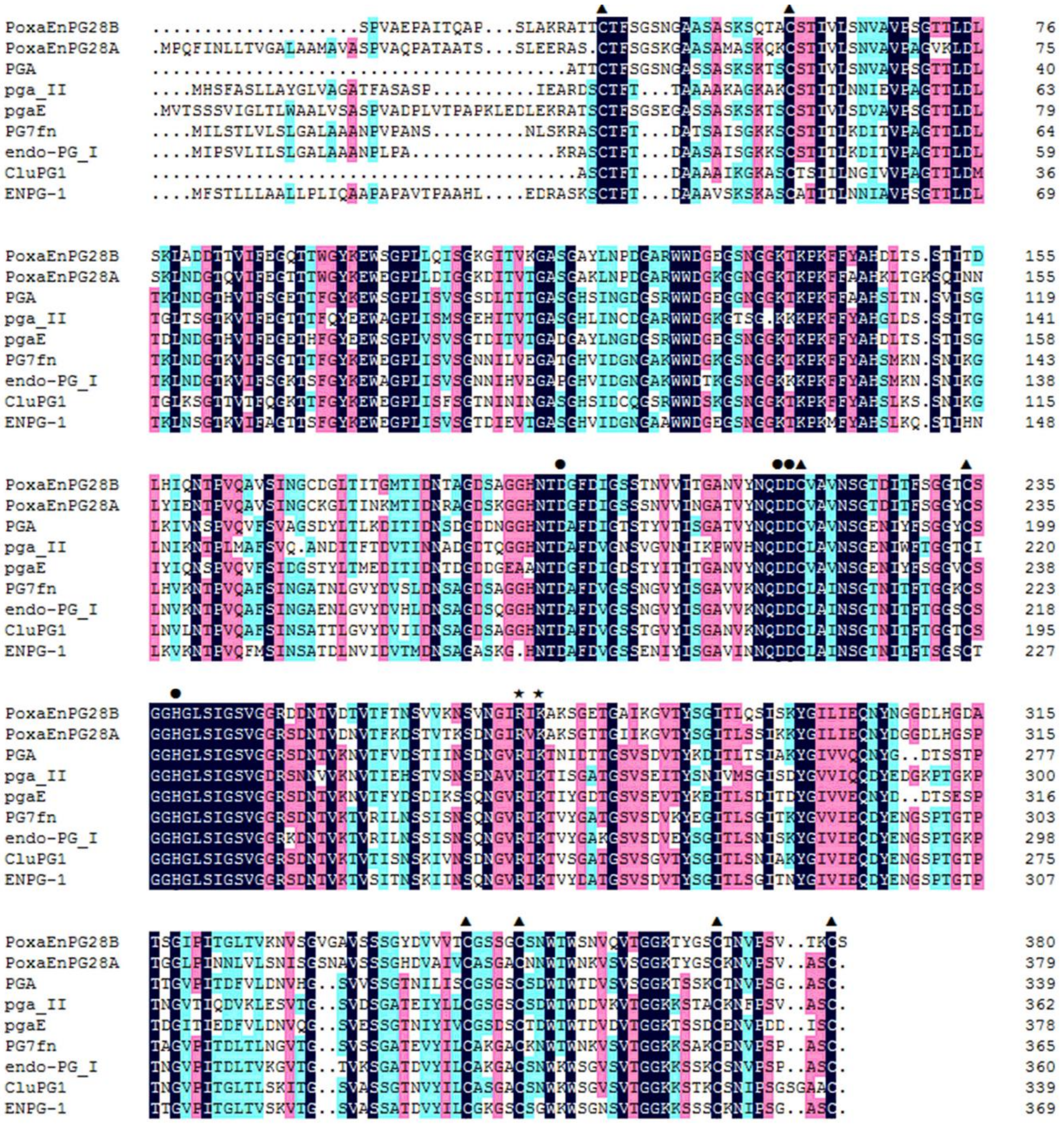

FIGURE S3. Alignment of amino acids of PoxaEnPG28B with reported fungal endo-PGases. The amino acid sequence encoded by the amplified cDNA was aligned with reported endo-PGases. Amino acid residues showing identities of 100%, higher than 75% and 50% were shaded in deep blue, light red and light green, respectively. The conserved amino acids Asp^{195}, Asp^{216}, Asp^{217} and His^{238}, which were predicted to play a crucial role in the catalytic function of the encoded protein, were marked by solid circles. Arg^{274} and Lys^{276}, which were predicted to be

involved in substrate binding, were marked by solid stars. Four predicted strictly conserved disulphide bridges (Cys^{40}-Cys^{58}, Cys^{218}-Cys^{234}, Cys^{346}-Cys^{351}, and Cys^{370}-Cys^{379}) were marked by solid triangles.

TABLE S1. Specific primers for gene cloning of *PoxaEnPG28B*.

Expression host	Vector	Recombinant protein	Primer name	Primer sequence (restriction endonuclease site was underlined)
Escherichia coli BL21(DE3)	pET22b(+)	PoxaEnPG28B-Ec	BL21–repgBF	ATTACCATGGCGTCACCCGTCGCTGAGCCG (*Nco* I)
			BL21–repgBR	ACGAGAGCTCCTAGTGGTGGTGGTGGTGGTGAGAGCACTTGGTCACACTGG (*Sac* I)
Pichia pastoris GS115	pPIC9K	PoxaEnPG28B-Pp	GS115–repgBF	GACCTACGTATCACCCGTCGCTGAGCCG (*SnaB* I)
			GS115–repgBR	CGACCTAGGTCAAGAGCACTTGGTCACACTGG (*Avr* II)

TABLE S2. Purification of the recombinant polygalacturonase PoxaEnPG28B-Pp.

Purification step	Enzyme activity (U)	Protein (mg)	Specific activity (U/mg)	Purification fold	Yield (%)
Culture broth	221508.4	14.2	15599.1	1.0	100.0
Ultrafiltration	203146.0	7.8	26044.3	1.6	91.7
SEC	36770.5	3.3	31974.3	2.0	16.6

TABLE S3. Application of PoxaEnPG28A in fruit juices extraction at 65 °C.

Fruits	Enzyme dosages (mg/kg pulp)	Reduction of viscosity (%)[a]	Increment of light transmittance (%)[a]	Increment of yield (%)[a]
Papaya	0.01	2.9±0.2	19.6±1.4	7.9±0.3
	0.02	4.5±0.2	20.2±1.1	9.1±0.4
	0.04	6.6±0.3	23.2±1.8	10.0±0.4
Banana	0.01	13.1±0.9	1.9±0.1	1.0±0.1
	0.02	24.2±1.3	2.0±0.1	1.0±0.1
	0.04	35.6±1.9	5.1±0.3	2.7±0.2
Plantain	0.01	36.3±2.2	27.6±1.9	35.2±0.8
	0.02	55.5±2.8	31.6±1.9	42.2±2.5
	0.04	68.7±3.4	34.0±2.7	53.5±3.7

[a] All experiments were performed in triplicate and mean values were presented. The experiments were repeated three times and similar results were obtained.

References

Bonivento, D., Pontiggia, D., Matteo, A.D., Fernandez-Recio, J., Salvi, G., Tsernoglou, D., et al. (2008). Crystal structure of the endopolygalacturonase from the phytopathogenic fungus *Colletotrichum lupini* and its interaction with polygalacturonase-inhibiting proteins. *Proteins* 70(1), 294–299.

Bradford, M.M. (1976). A rapid and sensitive method for the quantitation of microgram quantities

of protein utilizing the principle of protein-dye binding. *Anal Biochem* 72(1–2)**,** 248–254.

Cheng, Z., Chen, D., Wang, Q.Y., Xian, L., Lu, B., Wei, Y.T., et al. (2017). Identification of an acidic endo-polygalacturonase from *Penicillium oxalicum* CZ1028 and its broad use in major tropical and subtropical fruit juices production. *J Biosci Bioeng* 123(6)**,** 665–672.

Cho, S.W., Lee, S., and Shin, W. (2001). The X-ray structure of *Aspergillus aculeatus* polygalacturonase and a modeled structure of the polygalacturonase-octagalacturonate complex. *J Mol Biol* 311(4)**,** 863–878.

Gao, S., Choi, G.H., Shain, L., and Nuss, D.L. (1996). Cloning and targeted disruption of enpg-1, encoding the major in vitro extracellular endopolygalacturonase of the chestnut blight fungus, *Cryphonectria parasitica*. *Appl Environ Microb* 62(6)**,** 1984–1990.

Gonzalez, C.F., Pettit, E.A., Valadez, V.A., and Provin, E.M. (1997). Mobilization, cloning, and sequence determination of a plasmid-encoded polygalacturonase from phytopathogenic *Burkholderia* (*Pseudomonas*) *cepacia*. *Mol Plant Microbe Int* 10(7)**,** 840–851.

Laemmli, U.K. (1970). Cleavage of structural proteins during the assembly of the head of bacteriophage T4. *Nature* 227(5259)**,** 680–685.

Liu, M.Q., Dai, X.J., Bai, L.F., and Xu, X. (2014). Cloning, expression of *Aspergillus niger* JL-15 endo-polygalacturonase A gene in *Pichia pastoris* and oligo-galacturonates production. *Protein Expres Purif* 94**,** 53-59.

Massa, C., Degrassi, G., Devescovi, G., Venturi, V., and Lamba, D. (2007). Isolation, heterologous expression and characterization of an endo-polygalacturonase produced by the phytopathogen *Burkholderia cepacia*. *Protein Expres Purif* 54(2)**,** 300-308.

Morán-Diez, E., Hermosa, R., Ambrosino, P., Cardoza, R.E., Gutiérrez, S., Lorito, M., et al.

(2009). The ThPG1 endopolygalacturonase is required for the *Trichoderma harzianum*–plant beneficial interaction. *Mol Plant Microbe In* 22(8), 1021–1031.

Pařenicová, L., Benen, J.A.E., Kester, H.C.M., and Visser, J. (1998). *pga*E encodes a fourth member of the endopolygalacturonase gene family from *Aspergillus niger*. *Eur J Biochem* 251(1–2), 72–80.

Rafique, N., Tabassum, R., Awan, M.S., Orts, W., and Wong, D.W.S. (2016). Cloning and expression of *Pectobacterium carotovorum* endo-polygalacturonase gene in *Pichia pastoris* for production of oligogalacturonates. *BioResources* 11(2), 5204–5214.

van Santen, Y., Benen, J.A.E., Schröter, K.H., Kalk, K.H., Armand, S., Visser, J., et al. (1999). 1.68-Å crystal structure of endopolygalacturonase II from *Aspergillus niger* and identification of active site residues by site-directed mutagenesis. *J Biol Chem* 274(43), 30474–30480.

Sieiro, C., Sestelo, B.F., and Villa, T.G. (2009). Cloning, characterization, and functional analysis of the EPG1-2 gene: A new allele coding for an endopolygalacturonase in *Kluyveromyces marxianus*. *J Agric Food Chem* 57(19), 8921–8926.

Tamura, K., Dudley, J., Nei, M., and Kumar, S. (2004). MEGA4: Molecular evolutionary genetics analysis (MEGA) software version 4.0. *Mol Biol Evol* 24(8), 1596–1599.

Tu, T., Meng, K., Bai, Y.G., Shi, P.J., Luo, H.Y., Wang, Y.R., et al. (2013). High-yield production of a low-temperature-active polygalacturonase for papaya juice clarification. *Food Chem* 141(3), 2974–2981.

Tu, T., Meng, K., Huang, H.Q., Luo, H.Y., Bai, Y.G., Ma, R., et al. (2014). Molecular characterization of a thermophilic endopolygalacturonase from *Thielavia arenaria* XZ7

with high catalytic efficiency and application potential in the food and feed industries. *J Agr Food Chem* 62(52), 12686–12694.

Wang, J.J., Zhang, Y.H., Qin, X., Gao, L.Y., Han, B., Zhang, D.Q., et al. (2017). Efficient expression of an acidic endo-polygalacturonase from *Aspergillus niger* and its application in juice production. *J Agr Food Chem* 65(13), 2730–2736.

Xian, L., Wang, F., Yin, X., and Feng, J.X. (2016). Identification and characterization of an acidic and acid-stable endoxyloglucanase from *Penicillium oxalicum*. *Int J Biol Macromol* 86, 512–518.

Yang, J., Luo, H.Y., Li, J., Wang, K., Cheng, H.P., Bai, Y.G., et al. (2011). Cloning, expression and characterization of an acidic endo-polygalacturonase from *Bispora* sp. MEY-1 and its potential application in juice clarification. *Process Biochem* 46(1), 272–277.

Yuan, P., Meng, K., Huang, H.Q., Shi, P.J., Luo, H.Y., Yang, P.L., et al. (2011). A novel acidic and low-temperature-active endo-polygalacturonase from *Penicillium* sp. CGMCC 1669 with potential for application in apple juice clarification. *Food Chem* 129(4), 1369–1375.

Zhou, H.X., Li, X., Guo, M.Y., Xu, Q.R., Cao, Y., Qiao, D.R., et al. (2015). Secretory expression and characterization of an acidic endo-polygalacturonase gene from *Aspergillus niger* SC323 in *Saccharomyces cerevisiae*. *J Microbiol Biotechnol* 25(7), 999–1006.
